# The role of *LSR* gene variants in early onset intrahepatic cholestasis: a case series with treatment options

**DOI:** 10.3389/fped.2025.1582769

**Published:** 2025-09-04

**Authors:** Sinja Ohlsson, Alex Zaufel, Natalia Qvartskhava, Friedrich Stock, Sophie Hinreiner, Hideo A. Baba, Benas Prusinskas, Ralf Kubitz, Boris Görg, Diran Herebian, Elke Lainka, Simone Kathemann

**Affiliations:** ^1^ Clinic for Pediatrics II, University Medicine Essen, Essen, Germany; ^2^ Department of Gastroenterology, Hepatology and Infectious Diseases, University Hospital Düsseldorf, Heinrich Heine University Düsseldorf, Düsseldorf, Germany; ^3^ Institute of Human Genetics, University Medicine Essen, Essen, Germany; ^4^ Center for Human Genetics, Practice for Human Genetics Hehr, Regensburg, Germany; ^5^ Institute for Pathology, University Medicine Essen, Essen, Germany; ^6^ Department of Pediatrics, Vilnius University Hospital Santaros Clinics, Vilnius, Lithuania; ^7^ Department of Gastroenterology and Oncology, Bethanien Hospital, Moers, Germany; ^8^ Department of General Pediatrics, Neonatology and Pediatric Cardiology, University Hospital Düsseldorf, Heinrich Heine Universität Düsseldorf, Düsseldorf, Germany

**Keywords:** infantile intrahepatic cholestasis, early onset intrahepatic cholestasis, *LSR* gene, pruritus, itching, microcephaly, progressive familiar intrahepatic cholestasis, IBAT inhibitor

## Abstract

We report on three children with novel variants in the lipolysis-stimulated lipoprotein receptor (*LSR*) gene with clinical presentation with early onset intrahepatic cholestasis and the main symptom being uncontrollable itching. Two patients showed dystrophy, short stature and microcephaly, whilst one patient had neurological developmental delay. LSR is one component of special tricellular tight junctions (tTJs) with expression in the liver and brain. We analyzed clinical data for all patients and performed multigene panel sequencing followed by Human Phenotype Ontology (HPO) based exome analysis, classifying the sequenced variants according to the American College of Medical Genetics and Genomics (ACMG) guidelines. We performed immunostaining on the liver cryosections. The lack of LSR expression in immunofluorescence of the patients’ liver tissue confirmed the pathogenicity of genetic variants. We analyzed bile acids (BA) and their derivatives by ultra-performance liquid chromatography-mass spectrometry (UPLC-MS) in two of the three patients, confirming disturbed bile salt secretion. We also describe the use of an ileal bile acid transport (IBAT) inhibitor in two patients with LSR-associated intrahepatic cholestasis for the first time. Both patients showed a good response to the therapy in terms of itch control. In conclusion, LSR-associated early onset intrahepatic cholestasis is a new and likely underdiagnosed disease. Patients with an unclear progressive familial intrahepatic cholestasis (PFIC)-like clinical picture should therefore undergo genetic testing of the *LSR* gene. Treatment with an IBAT inhibitor should be considered.

## Introduction

The diagnosis of progressive familial intrahepatic cholestasis (PFIC) currently includes 13 heterogeneous subtypes (PFIC 1-13) of genetic, non-obstructive cholestatic liver disease due to impaired bile composition or secretion. PFIC 1-13 is caused by variants in the *ATP8B1*, *ABCB11*, *ABCB4*, *TJP2, NR1H4, SLC51A, USP53, KIF12*, *ZFYVE19*, *MYO5B*, *SEMA7A*, *VPS33B* and *PSKH1* genes (1–6). The clinical symptoms vary depending on the type, with the main symptoms being cholestasis, itching and progressive liver fibrosis, through to end-stage liver disease in some types. Odevixibat (Bylvay®) received approval as a therapeutic option for symptom control in patients with PFIC in 2021, whilst Maralixibat (Livmarli®) has been approved since 2024. Odevixibat and Maralixibat inhibit ileal bile acid transport (IBAT), thus promoting bile acid (BA) excretion via the intestine (7).

In addition to classic PFIC, four cases of PFIC-like infantile intrahepatic cholestasis (IIC) caused by mutation in the lipolysis-stimulated lipoprotein receptor (*LSR*) gene (*616582) have been described to date (4, 8). The patients conformed to the clinical picture of PFIC with cholestasis and uncontrollable itching as the main symptom, with onset in early childhood. Two patients were described with neurological developmental delay and three of them with microcephaly (4, 8).

LSR is one component of special tricellular tight junctions (tTJs) that form tricellular cell contacts between epithelial cells. As an integral membrane protein, human LSR comprises 581 amino acids and contains an extracellular immunoglobulin G (IgG) domain, and transmembrane and cytoplasmatic domains. In their study, Masuda et al. (9) proposed LSR to act as a landmark to recruit other molecular components such as tricellulin to the tTJs. LSR is expressed in the fetal and adult liver as well as in ovaries, testis, the adrenal gland, lung, intestine, kidney and brain. Mesli et al. (10) also showed that mice with two inactivated *LSR* alleles (LSR-/-) displayed embryonic lethality, liver hypoplasia and reduced cellular density.

## Methods

### Patient data

We report on three children with variants in the *LSR* gene and a PFIC-like clinical picture. All patients receive, or have received, inpatient and outpatient care at Essen University Children's Hospital. The clinical data (medical history, examination results, laboratory parameters, and genetic and histopathological findings) have been collected since September 2023 from existing patient files after obtaining the consent of the legal guardians. All blood and tissue samples from patients used in the study were taken as a part of regular clinical diagnostics and according to clinical indications for unclear cholestatic liver disease. Consent forms for genetic testing and blood and tissue sampling were available. The parents gave their consent for publication of the data.

### Massive parallel sequencing and sanger sequencing

Multigene panel sequencing followed by Human Phenotype Ontology (HPO) based exome analysis was carried out for all patients. Exome sequencing was performed followed by virtual panels. In cases where only the *LSR* gene was examined, virtual single-gene analysis was performed. Genomic deoxyribonucleic acid (DNA) was processed using the Nextera DNA Flex Exome enrichment protocol (Illumina, Inc., San Diego, California, USA) and sequenced on a NextSeq^TM^500 system (Illumina, Inc. San Diego, California, USA). All reads were aligned to the human reference genome (hg 19) and variant detection was performed by the SeqNext module (JSI Medical Systems GmbH, Ettenheim, Germany) software tool, which was used to filter and categorize all variants found. Novel sequence variants were further evaluated using AlamutVisualPlus (Interactive Biosoftware, Sofia Genetics, Saint-Sulpice-des-Rivoires, France) as well as public databases (gnomAD, ClinVar, HGMD and Decipher). After *in silico* characterization, the sequence variants were classified according to the American College of Medical Genetics and Genomics (ACMG) guidelines (12). Sanger sequencing was performed on an ABI 3100Dx XL Avant Sequencer using the ABI Prism Big-Dye^TM^ Terminator Cycle Sequencing Kit version 3.1 (Applied Biosystems, ThermoFisher Scientific, Waltham, Massachusetts, USA) according to the manufacturer's recommendations. This method was carried out to confirm sequence variants identified by multigene panel sequencing and for parental carrier testing.

### Immunofluorescence staining of liver cryosections

Liver biopsies were performed as ultrasound-guided percutaneous transhepatic core biopsies using MONOPTY Disposable Core Biopsy Instrument 16 G × 10 cm, 22 mm penetration, 1.7 cm length (BARD Biopsy Systems, Tempe, Arizona, USA). The histopathological sections were analyzed in our pathology department. Control pieces of normal human livers were surgically removed from patients with metastatic liver disease. Immunostaining of liver cryosections was performed as previously described (10). In brief, cryosections of human liver biopsies were immunostained with murine antibodies against multidrug resistance-associated protein 2 (MRP2; M2I-4, ALX-801-015), TJP1; 33-9100, Na+/K+ATPase (ATP1A1; clone C464.6, ZMS1029) and LSR; NBP1-89631 at a dilution of 1:50. Tissue samples were incubated with secondary antibodies conjugated to Alexa Fluor 488 (goat anti-mouse, A11029, green) or 546 (goat anti-rabbit, A11035, red) for 1 h at room temperature. Unless otherwise indicated, images were acquired with ZEN software (Ver.3.11) on an LSM 880 confocal laser scanning microscope (Zeiss, Jena, Germany) using Immersol 518 F (Zeiss) oil on a Zeiss Plan-APOCHROMAT 63 × oil/DIC objective at room temperature.

### Quantification of serum BA levels

BA and their glycine and taurine derivatives were analyzed by ultraperformance liquid chromatography-mass spectrometry (UPLC-MS/MS). The method has already been described in detail elsewhere (10). The system consisted of an Acquity UPLC-H Class (Waters, UK) coupled to a Xevo-TQS tandem mass spectrometer (Waters, Milford, Massachusetts, USA) which is equipped with an ESI source in the negative ion mode. Data were collected in the multiple reaction monitoring (MRM) mode.

### Ethics statement

This study involving human participants was reviewed and approved by the Ethics Committee of the Medical faculty of the University Duisburg-Essen (vote 25-12327-Bo). Written informed consent from the participants' legal guardian/next of kin was not required to participate in this study in accordance with national legislation and the institutional requirements.

## Results

### Case series

#### Patient 1

Female patient of Indian origin from non-consanguineous parents had presented with clinically relevant itching since the age of 6 months (Table 1). The physical and neurological development was unremarkable. Laboratory chemistry showed a significant increase in BAs and a mild increase in aminotransaminases and gamma GT with normal bilirubin. The liver biopsy revealed grade 2–3 fibrosis, and the immunohistochemical examination for known transporter defects (BSEP, MDR3, MRP2 and CD10/CD13) was negative. Previous multi gene panel testing revealed one heterozygous variant each in the *ABCB4* and *ABCB11* genes, which were each assessed as unclear variants (ACMG class 3). In addition, a heterozygous variant was found in the *CFTR* gene, which was assessed as a likely pathogenic variant (ACMG class 4). In the case of autosomal recessive inheritance for *CFTR, ABCB11* and *ABCB4* related disorders a second variant would be necessary to confirm the diagnosis, but this could not be detected. A whole exome sequencing performed in India revealed two variants in the *LSR* gene. These two *LSR* variants were therefore analyzed in the patient and her parents by Sanger sequencing. Two compound-heterozygous variants were found in the *LSR* gene (c.257A>T, p.(Asp86Va); c.1274dup, p.(Glu426Glyfs*43)), which were classified as likely pathogenic (class 4) according to ACMG guidelines.

**Table 1:** Summary of the clinical features and genetic findings in our three patients with infantile intrahepatic cholestasis (IIC) with variants in the LSR gene.

Patient characteristics	Patient 1, 2y	Patient 2, 9y	Patient 3, 2y
Gender	Female	Male	Female
Origin	India	Germany	Albania
Consanguinity	No	No	Yes
Itching	Yes	Yes	Yes
Symptomatic	Since 6th month of life	Since 2nd years of life	Since 8th month of life
Neurological development	Normal	Severe combined developmental delay, intelligence impairment, behavioral abnormality	Normal
Physical development	10th percentile for height and weight	Dystrophy, short stature, microcephaly	Dystrophy, short stature, microcephaly
Laboratory chemistry			
- bilirubin - g-glutamyltransferase - aminotransferase - bile acids	Normal Increased (1.5× standard value) Increased (2× standard value) Increased (23× standard value)	Normal Increased (2.5× standard value) Normal Increased (50× standard value)	Normal? Normal Increased (5–7× standard value) Increased (16× standard value)
Liver biopsy			
- IHC transporter deficiency - stage of fibrosis	Negative II–III°	Negative IV°	Negative I–II°
Variants *LSR*-Gene (NM_205834.4)	Compound-heterozygous c.257A>T c.1274dup	Compound-heterozygous (most likely) c.745C>T p.Cys249Arg c.496G>A p.Gly166Ser	Homozygous c.571C>T p.Leu191Phe
Classification (ACMG guidelines)	Class 4	Class 4	Class 3
Other variants	*ABCB4* (heterozygous) c.3193C>G, p.(Leu1065Val) = uncertain significance (class 3) *ABCB11* (heterozygous) c.1441G>A, p.(Val481Met) = uncertain significance (class 3) *CFTR* (heterozygous) c.1037T>C, p.(Leu346Pro) = likely pathogenic variant (class 4)	*GARS1* (heterozygous) c.2091delA, p.Glu698Argfs*8 *LIPT2* (heterozygous) c.54G>C, p.Glu18Asp c.433G>T, p.Val145Phe *TUBGCP6* (heterozygous) c.1193C>T, p.Ser389Leu c.4081 C>T, p.Pro1361Ser *ASH1l* (heterozygous) c.5093G>A, p.Gly1698Glu *KCNN4* (heterozygous) c.159+5G>C = each uncertain significance (class 3)	*ATP8B1* (heterozygous) Exon12 c.711T>C(p.A257A) Exon23 c.698+201C>T Intron c.3532-15C>T Intron c.3756+11C>T *BSEP* (homozygous) Exon12 c.1331T>G(p.V444A) Exon23 c.384A>G(p.A1028A) Intron c.909-15A>G Intron c.219-27C>G *MDR3* (ex.heterozygous, in.homozygous) Exon5 c.504C>T(p.N168N) Exon5 c.1759G>A(p.R590Q) Intron c.2211+16C>T Intron c.3508-16T>C *ABCB4* (heterozygous) c.1769G>A,p.(Arg590Gln) = each uncertain significance (class 3)

Pedigrees are provided in Supplementary Figure S1. Conventional itching treatment for liver-related itching, such as colestyramine, ursodeoxycholic acid, rifampicin, naltrexone, phenobarbital and ondansetron, did not alleviate symptoms. Treatment with Odevixibat (Bylvay®) was initiated at the age of 2 years following the diagnosis of IIC. Follow-up was possible for 9 months until the patient moved back to India. A convincing response with a significant reduction of itching was seen after just 5 days and within the whole follow-up period. No relevant side effects were observed. As it is a genetic defect, the family planned a curative liver transplantation in India through a living donation from the father.

#### Patient 2

Male patient of German origin from non-consanguineous parents has presented with clinically relevant pruritus since the age of two. The patient has suffered from recurrent infection-associated seizures since the postnatal period. He has also displayed severe combined developmental delay, intellectual disability and behavioral problems, and in addition has dystrophy, short stature and microcephaly. Laboratory chemistry showed a significant increase in BA and a mild increase in gamma GT with normal bilirubin. The liver biopsy revealed liver cirrhosis but the immunohistochemical examination for known transporter defects was negative. Previous multi gene panel testing for genes associated with cholestasis and further testing revealed unclear heterozygous unclear variants. Based on the findings in patient 1, we also performed a targeted examination of the *LSR* gene. Two heterozygous variants were found in the *LSR* gene (c.745C>T p.Cys249Arg, c.496G>A p.Gly166Ser), which were classified as likely pathogenic (class 4) according to ACMG guidelines. Based on the clinical manifestation we suspect that both variants are compound heterozygous. However, as the father could not be examined, this cannot be stated conclusively. Pedigrees are provided in Supplementary Figure S1. Since the boy had refused all medication, pruritus therapy had not been possible until after the installation of a percutaneous endoscopic gastrostomy, after which medication could be administered for the first time. At age 9 years, the patient was treated with Odevixibat (Bylvay®) for 7 months and demonstrated a significant reduction in pruritus, with an accompanying improvement in quality of life, which continues to date. In addition, no relevant side effects were observed. Unfortunately, after 7 months, the gastric tube became blocked by Odevixibat, which dissolves only poorly, so that the patient was switched to the liquid drug Maralixibat (Livmarli®).

#### Patient 3

Female patient of Albanian origin from consanguineous parents has presented with clinically relevant itching and elevation of aminotransferases since the age of 8 months. The neurological development has so far been unremarkable, but the patient shows dystrophy, short stature and microcephaly. Laboratory chemistry shows a mild increase in BA but with normal bilirubin and gamma GT. The liver biopsy revealed grade 1–2 fibrosis and the immunohistochemical examination for known transporter defects was negative. Previous multi gene panel testing for genes associated with PFIC and congenital defect in bile acid synthesis revealed one heterozygous variant in the *ATP8B1* gene, one heterozygous variant in the *ABCB4* gene, one homozygous variant in the *BSEP* gene and a variant in *MDR3* gene, which were each assessed as unclear variants (ACMG class 3). Based on the findings in patient 1, we also performed a targeted examination of the *LSR* gene. One homozygous variant (c.571C>T p.Leu191Phe) was found. Since the parents (both heterozygous for the variant) are consanguineous, the variant can only be classified as unclear variant (class 3) according to ACMG guidelines. Pedigrees are provided in Supplementary Figure S1. At present, the itching is only mild and barely affects the patient's quality of life. An IBAT-inhibitor has therefore not been used to date, with only ursodeoxycholic acid prescribed as a regular medication. Rifampicin is given on demand during rare episodes of extreme itching.

### Detection of LSR in human liver by immunofluorescence

**Figure 1:**
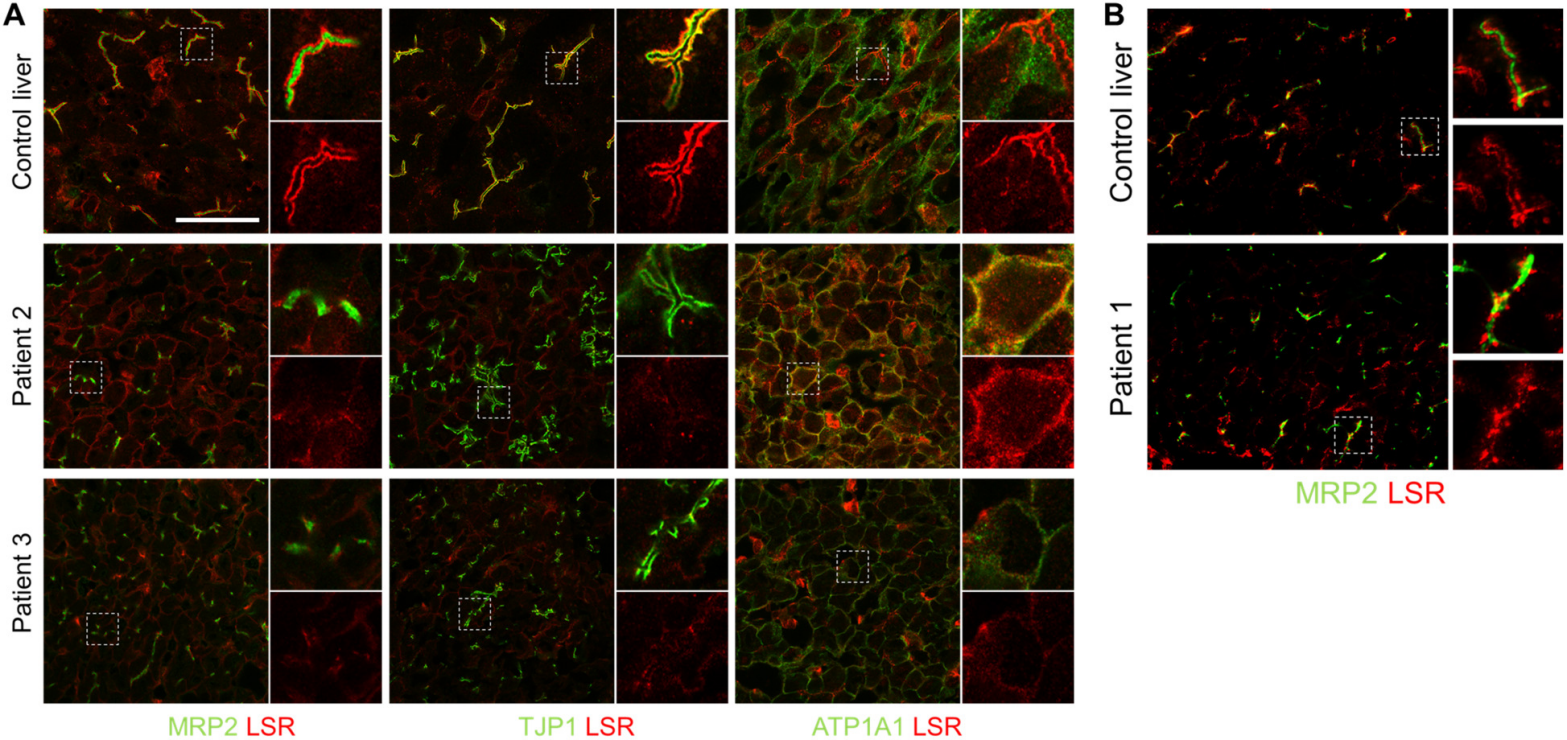
Immunofluorescence staining of LSR in liver slices. In normal human liver tissue (control liver), LSR (red), MRP2, TJP1, and ATP1A1 (green) were all strongly expressed and LSR colocalized at canalicular tight junctions with TJP1. **(A)** In the livers of both patients 2 and 3, TJP1 immunofluorescence (green) was largely preserved, while LSR immunoreactivity (red) was markedly reduced and colocalized aberrantly with ATP1A1 (green) at the basolateral plasma membrane. **(B)** In the liver of patient 1, MRP2 immunofluorescence (green) was preserved, while LSR immunoreactivity (red) was markedly reduced. There was insufficient patient material available for a complete set of immunofluorescence stains as shown in **(A)** Images in **(B)** were acquired with ZEN Blue 3.5.093 imaging software (ZEISS) on an Axio Observer.Z1 microscope with a C-Apochromat 63×/1.20 W Korr UV VIS IR objective. Scale bar, 50 μm. LSR, lipolysis-stimulated lipoprotein receptor; MRP2, multidrug resistance-associated protein 2; TJP1, tight junction protein 1; ATP1A1, ATPase Na(+)/K(+) transporting subunit alpha 1.

To investigate the pathophysiological relevance of these newly discovered mutations, liver biopsies were co-stained with LSR and specific antibodies. In normal human liver tissue, LSR surrounds the bilirubin transporter MRP2 within the canalicular membrane and co-localizes with TJP1 at the canalicular tight junctions to display a distinct immunoreactivity. LSR was still detectable in the livers of all three patients (Figure 1), although to a greatly reduced extent. However, while the staining of MRP2 and TJP1 was mostly preserved and localized to the canalicular membrane and canalicular tight junctions, respectively, LSR was predominantly aberrantly located at the basolateral plasma membrane, as shown by co-staining with ATP1A1 in patients 2 and 3. The detection of aberrantly located LSR without gross disruption of the bile canaliculus therefore confirms that LSR is of central importance for the physiological functions of the bile canaliculus.

### Bile salt analysis confirms disturbed bile salt secretion

**Figure 2:**
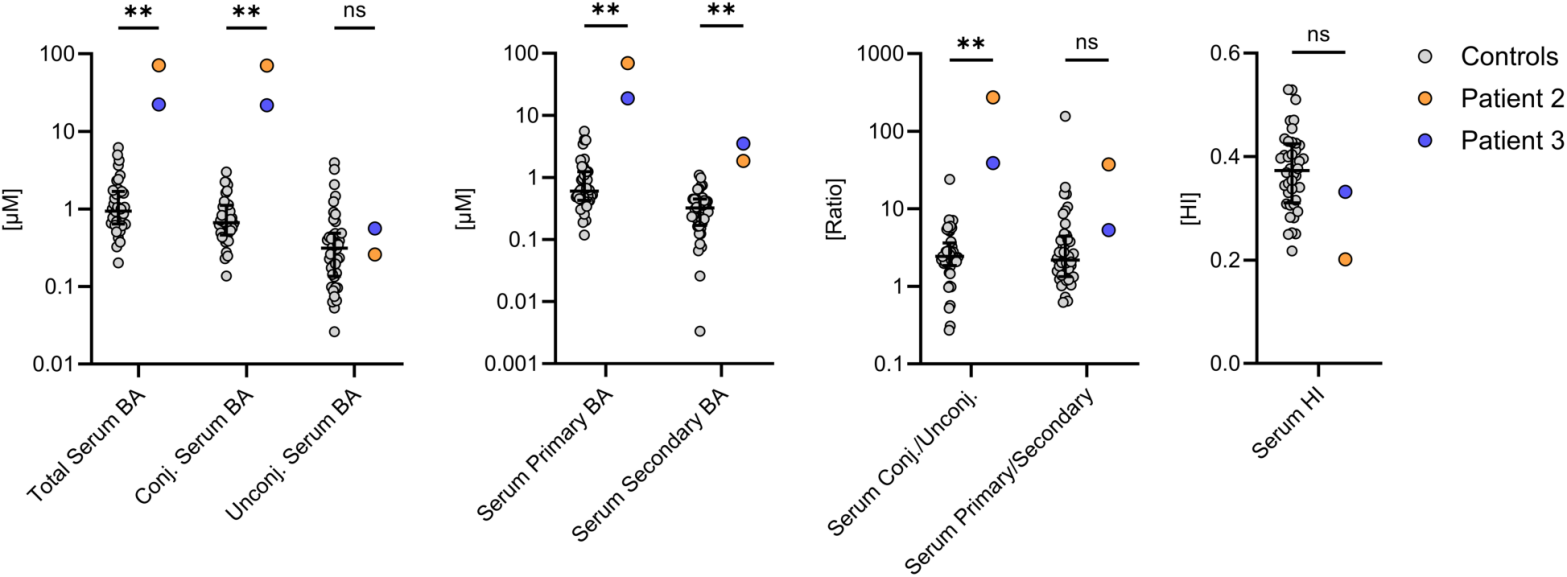
Serum bile acid (BA) composition in patients 2 and 3. Both patients had significantly increased serum BA concentrations, which was mainly due to a disproportionate increase in primary and conjugated BA. The BA profiles were compared with the measurements of 40 control subjects (grey). *P < 0.05, **P < 0.01. Statistical significance was calculated using an unpaired Mann–Whitney test. Data are depicted as median with interquartile range. BA, bile acids; HI, hydrophobicity index.

Primary and secondary BA and their taurine and glycine conjugates were analyzed from the sera of patient 2, patient 3 and 40 controls by high performance liquid chromatography-mass spectrometry (HPLC-MS/MS) (Figure 2). Although both patients showed no or only slight elevations of serum bilirubin and gamma GT, total serum BA were significantly elevated to 71.0 µM in patient 2 and 22.3 µM in patient 3 (normal median: 0.94 µM, *n* = 40). Unconjugated and secondary BA, which are produced by bacterial deconjugation and dihydroxylation in the large intestine, were still present in both patients. However, the increased total serum BA concentrations were mainly due to a disproportionate increase in conjugated and primary BA concentrations, as indicated by the increased ratios between conjugated/unconjugated and primary/secondary BA. The hydrophobicity index tended to be decreased in both patients, suggesting that BA overload rather than toxic BA composition contributed to liver injury in patients 2 and 3. Thus, these data indicate that both patients excrete insufficient amounts of BA, but without complete disruption of the enterohepatic circulation.

## Discussion

We report on three children with early onset intrahepatic cholestasis caused by novel variants in the *LSR* gene. Based on the ACMG guidelines, the genetic findings of our patients were classified as likely pathogenic (patient 1 and 2) and variant of unknown significance (patient 3), respectively. The clinical characteristics of these children and the lack of LSR expression in the immunofluorescence of the patients’ liver tissue confirm the pathogenicity of the genetic variants. All patients had additional variants in other genes. Although no variants were described as primary causes for cholestasis and itching, they are potential confounders. Only four patients with LSR-associated early onset intrahepatic cholestasis have been reported to date (4, 8). These patients showed PFIC-like disease presentation, with itching being the main symptom. As with the other PFIC diseases, our patients showed a sub-individually variable clinical picture ranging from mild symptoms without neurological impairment to the full clinical picture with therapy-refractory itching, liver cirrhosis and severe neurodevelopmental delay. Previous data report on onset of symptoms in infancy (4, 8). Our case series shows that the obvious onset of symptoms does not necessarily have to be in infancy, but is rather a diagnosis of the early childhood with variable courses. As in the cases described above, one of our patients had severe neurological symptoms (combined neurodevelopmental delay, intellectual disability, behavioral problems, seizures) and two had microcephaly. Since expression of the LSR protein has also been detected in the brain, a neurological manifestation of the diseases is plausible. However, further research is required as to whether the variants in the *LSR* gene are the exclusive the cause of the neurological symptoms. Nevertheless, the neurological development of patients with *LSR* variants should receive close monitoring, whilst long-term observation is necessary to better assess any prognosis. Further biochemical investigations into the function of the LSR protein in the liver - but also in other tissues such as the brain - should be performed for a better understanding of the pathogenesis of this disease. Masuda et al. (9) previously described LSR as an integral component of tTJs to form tricellular cell contacts between epithelial cells. The pathogenesis may be similar to that of PFIC 4, in which the cell contacts in the canalicular membrane and between the cholangiocytes are disrupted due to deficiency of TJP2. As with LSR, TJP2 is also expressed in multiple tissues. In the pathomechanism of PFIC 4, it is assumed that the BA themselves additionally stress the cell-cell contacts, so that they are even less stable than in other tissues (11). This explanation would also be reasonable be for LSR-associated early onset intrahepatic cholestasis.

In our study, we describe the use of an IBAT inhibitor in two patients with LSR-associated early onset intrahepatic cholestasis for the first time. Both patients showed a good response to the therapy in terms of itch control. No side effects were observed. Although the long-term results of its use remain to be seen, and the number of cases is small, the use of an IBAT inhibitor appears to be promising. Odevixibat and Maralixibat may delay or prevent liver transplantation and be a conservative treatment for pruritus.

## Conclusion

In summary, *LSR*-associated early onset intrahepatic cholestasis a new and probably underdiagnosed disease. Patients with an unclear PFIC-like clinical picture should therefore undergo genetic testing of the *LSR* gene. More genetic testing will expand the etiology and understanding of PFIC-related diseases and bile transport.

## Data Availability

The raw data supporting the conclusions of this article will be made available by the authors, without undue reservation.

## Supplementary material

Supplementary Figure S1 Immunofluorescence staining of LSR in liver slices of patient 1.
