# Supplementary figures and images for: The role of LSR gene variants in early onset intrahepatic cholestasis: a case series with treatment options
Source: Front Pediatr. 2025 Sep 4;13:1582769. doi: 10.3389/fped.2025.1582769 (PMC12443775; doi:10.3389/fped.2025.1582769)

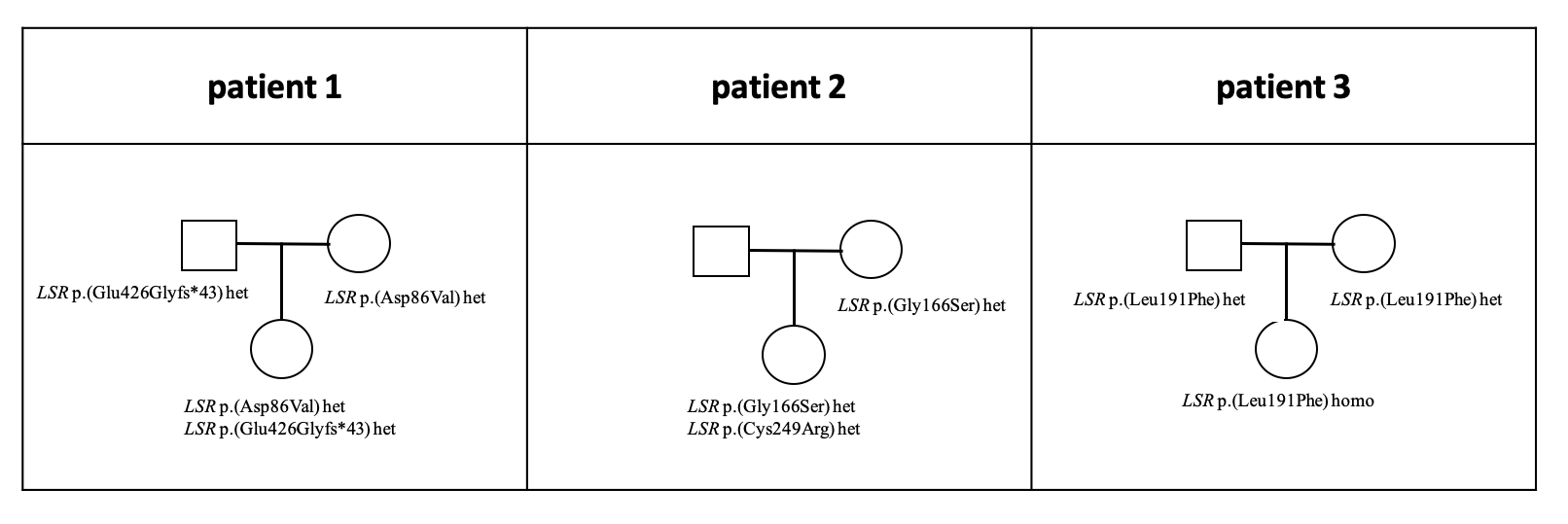

Supplement: Supplementary Figure S1 — Immunofluorescence staining of LSR in liver slices of patient 1. [file Image1.png]
